# A composite of platelet-like orientated BiVO_4_ fused with MIL-125(Ti): Synthesis and characterization

**DOI:** 10.1038/s41598-019-46498-w

**Published:** 2019-07-11

**Authors:** Philani Vusumuzi Hlophe, Lwazi Charles Mahlalela, Langelihle Nsikayezwe Dlamini

**Affiliations:** Department of Chemical Sciences, University of Johannesburg, Doornfontein Campus, P.O. Box 17011, Doornfontein, Johannesburg 2028 South Africa

**Keywords:** Photocatalysis, Synthesis and processing

## Abstract

The development of heterojunctions is the current focus of the scientific community as these materials are visible light active and the staggered positioning of their band edges combats electron-hole recombination which is the downside of most photocatalysts. In this work, a two- step hydrothermal synthesis protocol was utilized to fabricate a novel observable-light active material, composed of platelet-like BiVO_4_ and a titanium-based metal organic framework (MOF) called MIL-125(Ti). The tuning of specific morphologies, such as platelet-like in BiVO_4_, provides the exposure of most reactive facets which are more reactive towards photooxidation of organics in water, thus increasing their efficiency. The as-synthesized heterojunction was characterized by Transmission electron microscopy (TEM), scanning transmission microscopy (SEM), X-Ray diffraction (XRD), Raman spectroscopy, ultraviolet-visible diffuse reflectance spectra (UV-Vis DRS), X-Ray photoelectron spectroscopy (XPS) and photoluminescence (PL) spectra. The formation of the heterojunction lead to a positive shift of the 3-2 Bi:Ti valence band (VB) (1.78 eV) when compared to 1.27 eV VB position of BiVO_4_. The PL and photoelectrochemical measurements revealed that the heterojunction photocatalyst designated 3-2 Bi-Ti demonstrated inhibited recombination rate (platelet-like BiVO_4_ > 3-2 Bi:Ti (PM) > MIL-125 > 1–1 Bi:Ti > 2–3 Bi:Ti > 3-2 Bi:Ti) and highly efficient interfacial charge shuttle between platelet-like BiVO_4_ and MIL-125(Ti) through the formed *n*-*n* junction.

## Introduction

Several photocatalyst uses have been reported in literature and they include uses such as for storage of energy, sterilization, self-cleaning, air purification and wastewater treatment^1,2^. Work in these areas have been vastly explored using TiO_2_ as the base photocatalyst^3^. The application of TiO_2_ is only limited to UV absorption, due to its large band gap, which leads to low efficiency for the visible spectrum and high recombination rate^4^.

Even though there are available visible light active photocatalysts that have been used in photodegradation, such as BiVO_4_ and WO_3_, they further face an issue with fast electron-hole pairs recombination^5,6^. This fast recombination of the electron-hole pairs allows only for few electrons and holes to migrate to the surface and participate in catalytic reactions on the surface of the photocatalyst which results in poor efficiency thus stagnating industrial application^7^.

The synthesis of composite materials with the aim to formulate a heterojunction has been explored as one of the important strategies for the separation of electron-hole pairs in photocatalytic semiconductor materials. Heterojunction formation is a spatial separation technique which is band-edge offset dependant. These band-edge offsets separate electrons and holes by directing these charge carriers to different materials via a junction^8^. This allows the electrons and holes to spend more time apart, enough for reduction and oxidation to occur^7^.

Considerable attention has been given to bismuth vanadate (BiVO_4_) due to its narrow band gap of 2.4–2.5 eV, low-cost, nontoxicity, and favourable valence band position. These characteristics have enabled BiVO_4_ to emerge as a promising candidate for photoelectrochemical (PEC) water splitting and photocatalytic degradation of organic pollutants^9^. BiVO_4_ is intrinsically an *n*-type semiconductor that has a valance band position that is appropriate for visible-light activity^10^. Crystal polymorphs of BiVO_4_ include; zircon structure with a tetragonal system (z-t), scheelite structures with monoclinic (s-m) and tetragonal (s-t) phases^11^. Among these three phases, the monoclinic phase has been reported to be thermodynamically stable and demonstrates good photoactivity properties^9^.

Even though BiVO_4_ has shown good visible light activity, it suffers from slow electron transportation which is linked to fast recombination of photogenerated electron-hole pairs. Many attempts have been made to combat this through co-catalyst modifications to form heterojunctions or phase-junctions, doping with non-metals or metals such as Fe, W, N, P and Mo, and tuning morphology with the aim to expose most reactive facets^10^. Doping involves the introduction of impurity levels that act as electron or hole traps thus facilitation separation of electrons and holes^12,13^. Co-catalyst modifications to form heterojunctions or phase-junctions depends on the band-edge staggered alignment that facilitate the shuttle of electrons and holes to different materials by separating the respective charge transporters. This has been made possible by using FeVO_4_, Bi_2_O_3_ and TiO_2_ to form heterostructures with BiVO_4_^14–16^. While phase junctions have been synthesized through the formation of monoclinic and tetragonal phases co-existing in one BiVO_4_ sample for spatial separation of electron-hole pairs through band-edge offsets resulting from the differences band positions of the different phases^17^.

BiVO_4_ is known to suffer from low surface area, thus in this work platelet-like BiVO_4_ was synthesized in hyperbranched polyethyleneimine (HPEI) to improve surface area and produce smaller nanoparticles. Facet design in BiVO_4_ has shown that the 010 facet is the most reactive and provides spatial separation of electrons and holes by increasing the availability of electrons on its surface while preventing electron hole recombination^18^. Tan *et al*. illustrated higher photooxidation activity of {010} facet of monoclinic BiVO_4_ in relative exposure extents with {110} facet, where the {110} traps holes while the electrons are trapped by the largely exposed {010} facet^18^. Huang *et al*. reported a correlated high activity to {010} facets of BiVO_4_ for the evolution of O_2_^19^. The novelty of this work is the tuning of the most reactive facet of BiVO_4_ whilst also using HPEI as a template for the development stable nanoparticles.

Hyperbranched polyethyleneimine belongs to a class of materials identified as dendrimers as it a branched macromolecule which consists of a diaminoethane core, interior branching polyethyleneimine intermediates and an abundance of surrounding NH_2_ functional groups which make the molecule hydrophilic^20,21^. The interior branching units render multiple internal nanocavities which can be utilized as a host for the production of small and robust nanoparticles because HPEI has a quasi-spherical structure that provides a shell which inhibits the aggregation of nanoparticles^21^.

Metal–Organic Frameworks (MOFs) are a category of translucent substances that comprise of coordinate bonds between metal nodes and organic ligands^22^. MOFs have come to the fore as a comprehensive set of crystalline materials with well-defined porosity. MOFs are different from other permeable materials, they boast many merits such as well-defined porosity, large surface area, ease of synthesis, thermal stability, ultra-low densities and with extensive properties convenient for physical and chemical implementation^23^. These intriguing features have triumphed the implementation of MOFs in gas storage, catalysis, segregation of fluids^24^. However, the limitation of these materials is the lack of visible light activation, which limits their efficiency upon photon irradiation.

Recently research focus has been on the photoactive, crystalline and highly porous titanium oriented MOFs including MIL-125(Ti) exhibiting Ti_8_O_8_(OH)_4_(BDC)_6_ BDC = 1,4-benzenedicarboxylic acid has garnered tremendous recognition in the photocatalysis domain^25^.

MIL-125(Ti) is a crystal clear titanium dicarboxylate exhibiting outstanding absorbance ability, encapsulation potential and good permeability, thermal strength and interesting photochemical characteristics^22^. As a result of the frameworks porosity, the secondary building units (SBUs) transport reductive intermediates away from the reactive sites. This is because the material has a rigid coordination environment ideal to create chromophore sites by introducing metal co-catalysts to the SBUs. Additionally this material is decorated with numerous inactive Ti sites^22^. However, its application in photocatalytic platforms is limited due to its activation only in the UV light region and fast charge recombination, resulting in poor photocatalytic efficiency. Not much work has been reported on fusing MIL-125(Ti) and facet controlled BiVO_4_ composite, with the potential of being used in various photocatalytic applications such as environmental remediation. Thus, in this work we report the combination of platelet-like BiVO_4_ with MIL-125(Ti) into a novel heterojunction photocatalyst which will enable the material to have minimum recombination and be photoactive in visible light.

## Materials and Methods

### Materials

The chemicals were used as received from manufacturers and they included; bismuth nitrate pentahydrate (98% reagent grade, Sigma-Aldrich Co.), ammonium metavanadate (≥99% ACS reagent, Sigma-Aldrich Co.), nitric acid (55%, Associated Chemical Enterprises), ethanol (≥99.8% absolute, Sigma-Aldrich Co.), ammonia solution (25% Associated Chemical Enterprises), nitric acid (55%, Sigma-Aldrich Co.), hyperbranched polyethyleneimine (Sigma-Aldrich Co.), sodium sulphate (≥99% ReagentPlus, Sigma-Aldrich Co.), polyvinylidene fluoride (GPC, Sigma-Aldrich Co.), *N*-methylpyridine (≥99% ACS reagent, Sigma-Aldrich Co.), Teraphthalic acid (BDC) (Sigma-Aldrich Co.), *N*^’^*N*-dimethylformanide (DMF) (Sigma-Aldrich), titanium isopropoxide (Sigma-Aldrich Co.) and methanol (Sigma-Aldrich).

### Synthesis of nanoparticles

Pristine MIL-125(Ti) was fabricated according to a synthesis route reported by Yang *et al*.^26^ with minor adjustments. Typically, 2.2 g of teraphthalic acid was dissolved in a solution of 36 mL DMF and 4 mL methanol. Titanium isopropoxide (2.4 mL) was then added to the resultant solution which resulted in a white solution. This was then sonicated for 5 mins to ensure a homogeneous mixture. The mixture was exposed to hydrothermal conditions in a 100 mL Teflon lined autoclave for 24 hr at 150 °C. Post cooling to room temperature, a gel-like solid was secured which was washed once with 50 mL DMF and twice with 50 mL Methanol. The solid was then dried for 24 hrs at 60 °C in air.

Platelet-like BiVO_4_ nanoparticles (NPs) were synthesized using a hydrothermal method with minor modifications as reported in one of our publications in the group^27^. Bismuth nitrate pentahydrate (4.90 g) and 1.20 g ammonium metavanadate were dissolved in 60 mL of 2 M nitric acid and the solution was stirred for 2 hrs until a yellow precipitate. In this solution, 3.75 g HPEI was then added, after which the pH was adjusted to pH 2 using 25% ammonia solution. NaCl (1.75 g) was added and the solution was stirred for 15 min. The solution was then subjected to hydrothermal conditions in a 100 mL Teflon lined autoclave and heated in an oven at 200 °C for 24 hrs.

Three heterojunctions of BiVO_4_/MIL-125(Ti) were synthesized via a coprecipitation method. The amounts in composites were based on Bi:Ti in the ratios 1:1, 2:3 and 3:2. The 3:2 composite was synthesized by measuring 0.972 g of platelet-like BiVO_4_ which was mixed by sonication with 0.50 g teraphthalic acid, 0.6 mL titanium isopropoxide, 9.0 mL DMF and 1.0 mL methanol for 5 min. The mixture was then subjected to similar hydrothermal conditions as those of pristine MIL-125(Ti) stated above. For the other composites, 0.648 g platelet-like BiVO_4_ was mixed with 0.80 g teraphthalic acid, 0.9 mL titanium isopropoxide, 13.3 mL DMF and 1.5 mL methanol for 2:3, while for 1:1, 0.324 g platelet-like BiVO_4_ was mixed with 0.30 g teraphthalic acid, 0.3 mL titanium isopropoxide, 0.45 mL DMF and 0.5 mL methanol. These were subjected to similar conditions as those of the 3:2 composite synthesis method. The composites were named as 1–1 Bi:Ti, 2–3 Bi:Ti and 3-2 Bi:Ti.

A physically mixed (PM) sample of the 3:2 ratio of Bi:Ti was also synthesized for the purpose of electrochemical impedance spectroscopy (EIS) characterization to give an insight on the formation of Schottky contacts in the hydrothermally synthesized heterojunction compared to physical mixing. This was necessary to also give proof that their formation in the hydrothermally synthesized materials enable the separation of electrons and holes compared to a physically mixed sample. Platelet-like BiVO_4_ (0.972 g) was mixed with 0.648 g of pristine MIL-125(Ti) using a pestle and mortar until a homogeneous mixture. This composite was named 3-2 Bi:Ti (PM).

### Characterization techniques

Morphologies of the NPs were captured using scanning electron microscopy (SEM) and transmission electron microscopy (TEM). The SEM (TESCAN Vega TC) was operated at 20 kV operating voltage under nitrogen gas using VEGA 3 TESCAN software. Elemental composition of the NPs was elucidated with the SEM coupled with EDS and was operated at 20 kV. A TEM which was operated at an accelerating voltage of 200 kV was used to further acquire images of the NPs. The NPs were deposited on carbon coated copper grids after being dispersed in ethanol. Selected area electron diffraction (SAED) was conducted on the NPs at 20–25 k magnification to elucidate their crystal phases.

Powdered X-ray diffraction (X’Pert Philips) with CuKα radiation (0.1540 nm) polychomator beam in the 2θ scan range 20–80 °C was carried to confirm the crystal phases of the NPs from the SAED patterns. A step size and step time of 0.0170 (2θ) and 87.63 s was used at 40 kV and 40 mA instrument power settings. Raman data were recorded for further confirmation of the crystal phases of the NPs. A Raman spectrometer, Raman Micro 200, Perkin Elmer precisely used with a single monochromator, a holographic notch and a cooled TCD. The samples were exposed for 4 s during excitation using the 514.5 nm Ar^+^ line. Optical properties were recorded from a diffuse reflectance spectroscopy (DRS) on a Shimadzu UV-2450 UV-Vis spectrophotometer. BaSO_4_ was used as a reference. Measurement of X-Ray photoelectron spectroscopy was carried out with a Thermo spectroscope, model ESCAlab 250Xi using a monochromator Al Kα (1486.7 eV) as an excitation source at a working pressure of <10^−8^ mBar. The photoluminescence spectra were recorded using a LS 45 fluorescence spectrometer (Perkin Elmer, precisely) at 242 nm wavelength.

Electrochemical measurements were conducted using Gamry IFC1000-11085 potentiostat in a standard three electrode system using Ag/AgCl as a reference electrode, a Pt wire as a counter electrode and a working electrode fabricated using fluorine-doped tin oxide (FTO) glass with NPs pasted on the fluorine-doped side. The working electrodes were prepared by mixing the synthesized NPs with polyvinylidene fluoride (PVDF) in a ratio 10:1 respectively in 1 mL N-methylpyridine (NMP). The PVDF was used as a binder and the NMP as a solvent to form a homogeneous slurry. The slurry was then drop casted on the FTO glass and allowed to dry overnight at room temperature. Electrical connection to the potentiostat was facilitated by attaching a copper wire using silver paste which was allowed to dry in air for 24 hr at room temperature. The electrochemical impedance spectroscopy (EIS) spectra was conducted at a frequency range of 10 kHz to 0.1 Hz at an AC voltage of 10 mV rms vs E_ref_.

## Results and Discussion

**Figure 1:**
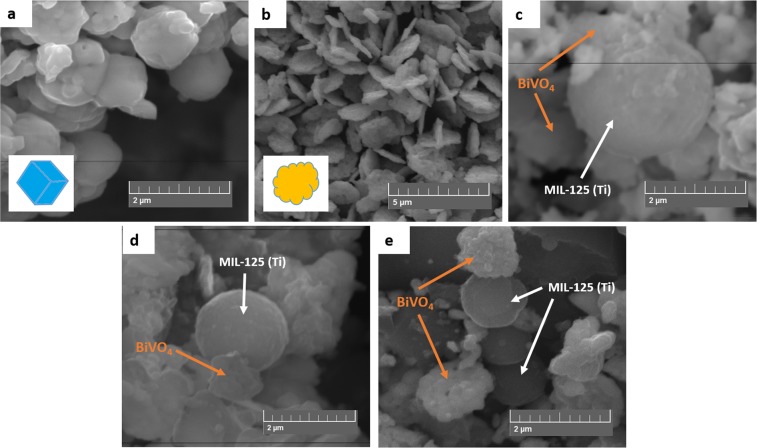
SEM images of (**a**) MIL-125(Ti), (**b**) BiVO_4_, (**c**) 1–1 Bi:Ti, (**d**) 2–3 Bi:Ti and (**e**) 3-2 Bi:Ti. The inserts in (**a**) and (**b**) are redrawn morphologies of the pristine MIL-125(Ti) and platelet-like BiVO_4_ respectively.

The images in Fig. 1 show the morphologies of the synthesized pristine materials and subsequent composites. More precisely, Fig. 1a represents the SEM image of MIL-125(Ti) which are rectangular shaped blocks. The SEM-EDS mapping confirmed the MIL-125(Ti) materials comprised of titanium, oxygen and carbon elements (Figure S1). BiVO_4_ NPs are shown in Fig. 1a which affirmed successful synthesis of platelet-like NPs. The SAED-EDS mapping in Figure S1b confirmed the presence of all the expected elements, mainly bismuth, vanadium and oxygen. Figure 1(c–e) represents the different composites fabricated which are 1–1, 2–3 and 3-2 Bi:Ti respectively. In all the composites the was an appearance of both the blocked and platelet-like NPs representing the presence of MIL-125(Ti) and BiVO_4_ NPs respectively. This showed the successful formation of the heterojunction materials. Further justification of the fabrication of the aforementioned materials is presented in Figs S3–5 (SEM-EDS mapping) which showed the presence of bismuth, vanadium, titanium, carbon and oxygen.

**Figure 2:**
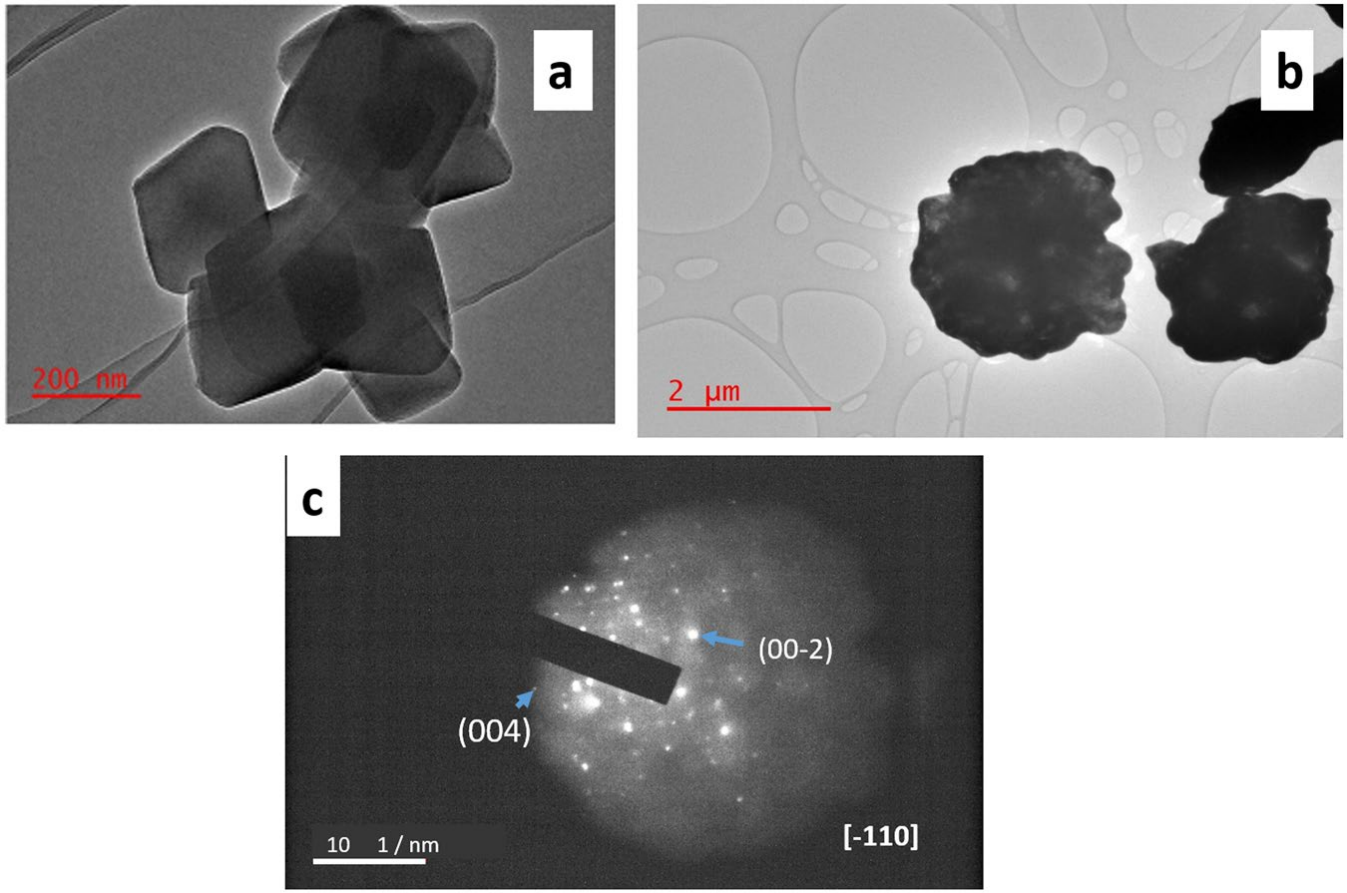
TEM images (**a**) MIL-125(Ti) (**b**) BiVO_4_. (**c**) SAED patterns of BiVO_4_.

**Figure 3:**
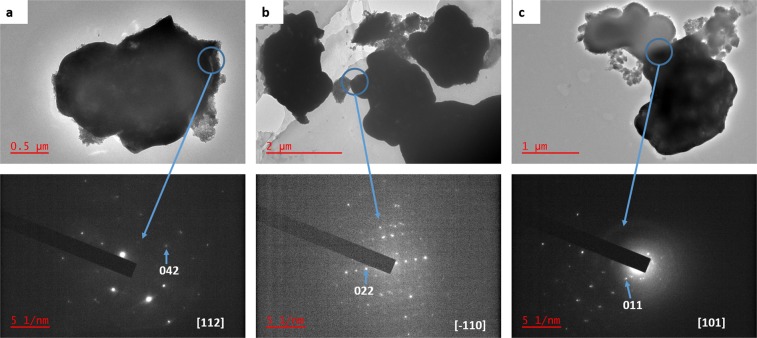
TEM images and respective SAED patterns of (**a**) 1–1 Bi:Ti, (**b**) 2–3 Bi:Ti and (**c**) 3-2 Bi:Ti composites of MIL-125(Ti)/BiVO_4_.

Figure 2 illustrates the TEM images of the pristine MIL-125(Ti) and platelet-like BiVO_4_ NPs. As evidenced by the SEM pictorial illustrations, the TEM confirmed the block and platelet-like NPs. The SAED patterns shown by Fig. 2c reveal the spots for platelet-like BiVO_4_ and this confirmed the synthesis of monoclinic BiVO_4_. The miller indices (042) and (002) shown are reported in literature to be those of monoclinic BiVO_4_. Composite material’s TEM images are depicted by Fig. 3(a–c) which confirmed the synthesis of the heterojunctions as the was an observation of both blocks and platelet-like NPs. In the 1–1 composite, the BiVO_4_ is shown to be deposited on the MIL-125(Ti). While for 2–3 and 3-2 some on the MIL-125(Ti) blocks were slightly attached to the platelet-like NPs. For all the composite materials, the observed SAED patterns confirmed monoclinic BiVO_4_ as the elucidated miller indices (042), (022), (011) in 1–1, 2–3 and 3-2 respectively are monoclinic spots. The were no record SAED patterns of the pristine MIL-125 as well as in the composite materials and none have been reported in literature which could mean it is not an SAED active material. Morphologies of the MIL-125(Ti) and BiVO_4_ were not distorted by the formation of the heterojunctions.

**Figure 4:**
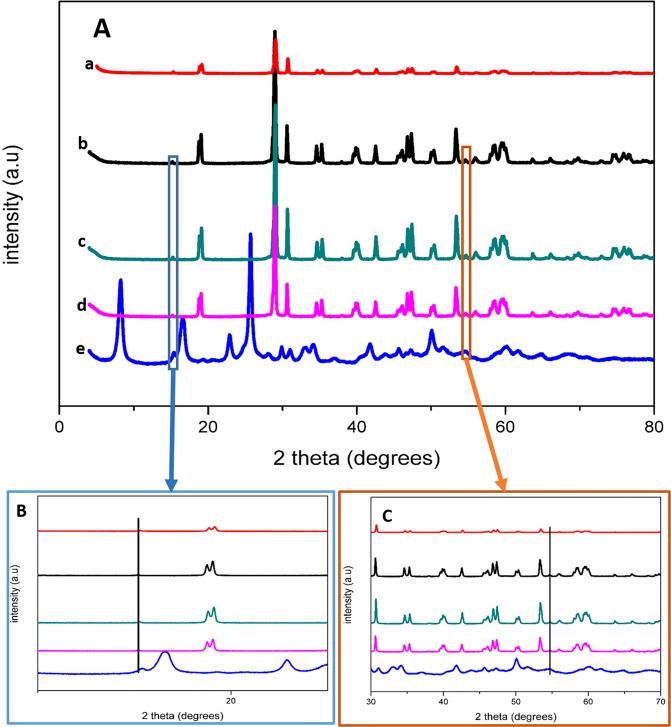
(**A**) XRD patterns of (a) BiVO_4_, (b) 1–1 Bi:Ti, (c) 3–2 Bi:Ti, (d) 2–3 Bi:Ti and (e) MIL-125(Ti). (**B**,**C**) are zoomed in images showing MIL-125(Ti) patterns in the composites.

Crystallographic studies were further elucidated on XRD illustrated in Fig. 4A and they confirmed monoclinic phases for platelet-like BiVO_4_ shown by (**a**). Monoclinic BiVO_4_ was also observed in the composite materials in (**b**-**d**) for 1–1, 3-2 and 2–3 Bi:Ti respectively. These results confirmed what was seen on the SAED patterns. These were indexed to the ICDD card 04-016-4328. Characteristic XRD patterns of MIL-125(Ti) were displayed in (e) which are consistent with literature^26,28–30^. This signaled that the MIL-125 Ti based MOF was successfully synthesized. An appearance of MIL-125(Ti) peaks were observed in all the fabricated composites as shown by the expanded regions in Fig. 1(a,c). The crystallinity of BiVO_4_ is much defined than that of MIL-125(Ti) which might be causing the BiVO_4_ patterns to be superior to those of MIL-125. This is similar to the work reported by Yang *et al*., BiVO_4_/MIL-125 showed a gradual weakening of MIL-125(Ti) patterns while patterns ascribed to BiVO_4_ were intensified, which suggested the dominance of BiVO_4_ in the composites^26^. This was a further affirmation of the successful hybridization of MIL-125(Ti) and BiVO_4_ composites and confirmed by SEM and TEM imagery.

**Figure 5:**
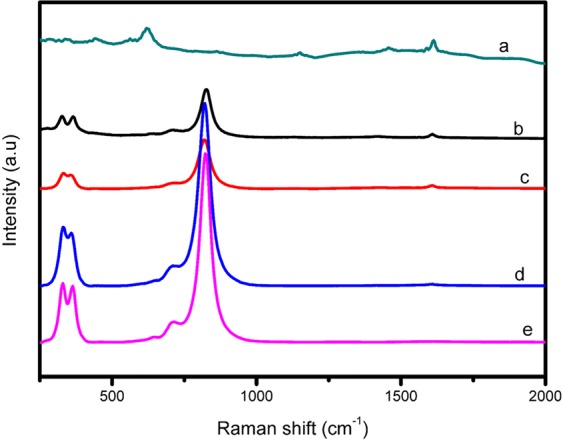
Raman spectra of (**a**) MIL-125(Ti), (**b**) 1–1 Bi:Ti (**c**) 2–3 Bi:Ti, (**d**) 3-2 Bi:Ti and (**e**) BiVO_4_.

Further investigation of the crystal phases was conducted by Raman spectroscopy (Fig. 5). The bands indexed at 634, 864, 1145, 1431, 1447 and 1617 cm^−1^ are characteristic of pristine MIL-125(Ti) in Fig. 5(a) and similar Raman bands were reported by Yuan and co-workers^22^. The bands are due to the vibration modes of the organic constituent H_2_BDC of this titanium based MOF^31,32^. Conclusively these bands correspond to the asymmetric and symmetric vibrational modes of the carboxylate group in addition to the vibrational modes of the C–H and C=C bonds of the benzene ring^22^. The variations of the Raman bands further confirmed the successful fabrication of MIL-125(Ti). Raman bands re-affirmed the monoclinic phase of platelet-like BiVO_4_ which complimented the XRD and SAED findings. Bands centred at 209, 323, 370, 708, and 830 cm^−1^ are representative of BiVO_4_^33^. Raman bands appearing at 830 and 708 cm^−1^ are ascribed to symmetric and asymmetric V–O stretching modes respectively. Those at 370 and 323 cm^−1^ are symmetric and asymmetric bending vibration of ${{\rm{VO}}}_{4}^{3-}$ tetrahedra respectively^33^. In the composite materials (Fig. 5(b–d)), the bands of both MIL-125(Ti) and monoclinic BiVO_4_ were observed. The band of MIL-125(Ti) at 1617 cm^−1^ was consistent in all the composites however it weakened with increasing amounts of BiVO_4_. The appearance of bands at 209, 323 and 830 cm^−1^ are indicative of monoclinic BiVO_4_ and were also consistent in all the composite materials. The presence of the mentioned bands of both MIL-125(Ti) and monoclinic BiVO_4_ confirmed the successful fabrication of the heterojunctions. Thus, the formation of the heterojunctions was concretely proved by SEM, TEM, XRD and Raman.

**Figure 6:**
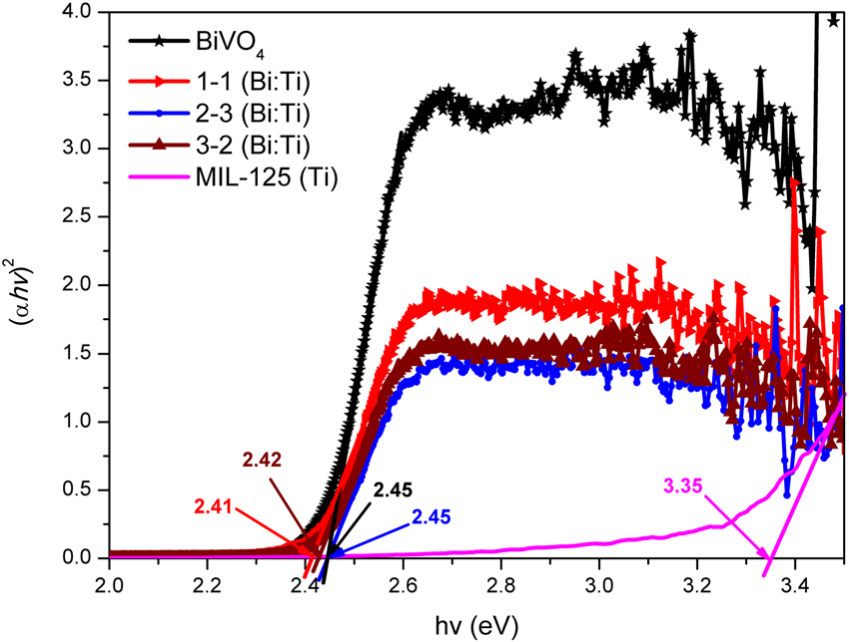
Tauc plots of the synthesized nanoparticles.

The optical properties of the NPs are shown in Fig. 6 and Figure S6. The band gap of the synthesized NPs, as indirect semiconductors, were calculated using Equation (1) using the DRS data^34^.

1 $$\alpha hv=A{(hv-{E}_{g})}^{n/2}$$

where α is the absorption coefficient, *h* is Plank’s constant, *v* is the incident light frequency, A is a constant, *E*_g_ is the band gap energy and *n* is 2. The computed band gaps from $hv(eV)=\frac{1240}{\lambda }$ on the (α*hv*)^2^ versus *hv* plots are shown in Fig. 6. A red shift of the band gaps was observed for the heterojunctions which is caused by the formation of the heterojunctions. It was also affirmed that platelet-like BiVO_4_ absorbs in the visible region^35^, while MIL-125(Ti) is UV active^26^. The band gaps of the pristine and composite materials are shown in Fig. 6.

**Figure 7:**
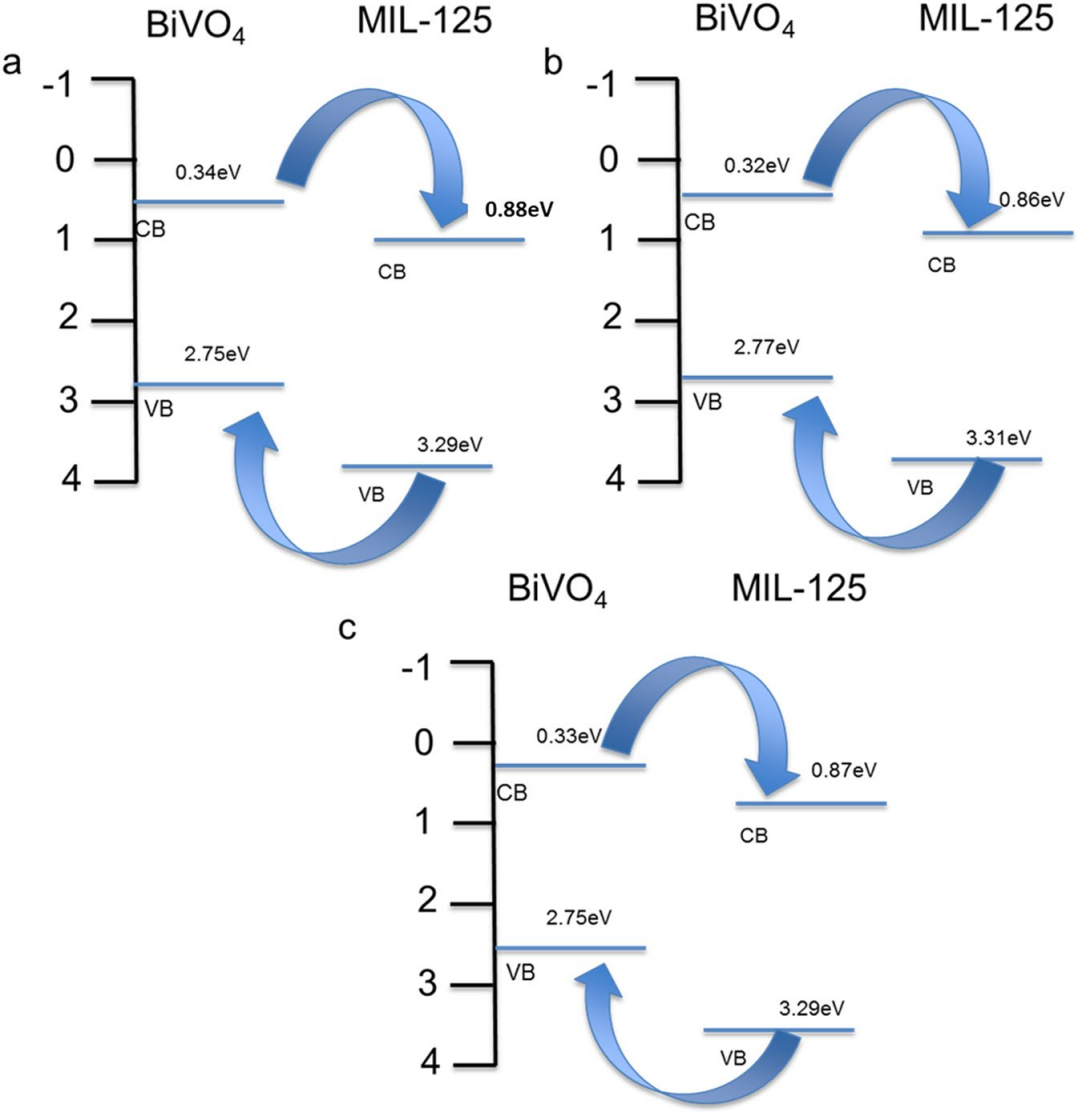
Band positions of the heterojunctions (**a**) 1–1 Bi:Ti, (**b**) 2–3 Bi:Ti and (**c**) 3-2 Bi:Ti.

The energy levels of the material were computed using the following empirical Equations (2 and 3):

2 $${E}_{VB}=X-{E}^{0}+0.5{E}_{g}$$

3 $${E}_{CB}={E}_{VB}-{E}_{g}$$

where *E*_*VB*_ and *E*_*CB*_ are the valence and conduction band (VB and CB) potentials respectively. Additionally *E*^0^ is the free electrons energy vs normal hydrogen electrode (*ca*. 4.5 eV) and *X* is the electronegativity of the semiconductor, expressed as the geometric average of the absolute electronegativity of the atoms^15^. The electronegativity of MIL-125(Ti) was computed to be approximately 6.58 eV, while that of BiVO_4_ was deduced from literature as 6.04 eV. The respective band positions are illustrated in Fig. 7 and they confirm successful formation of the heterojunctions for all the composites, specifically 1–1, 2–3 and 3-2 Bi:Ti in Fig. 7(a–c) respectively. Positioning of the CBs and VBs in the composites were well suited for the separation of electrons and holes during photoexcitation. This will allow the flow of electron from the platelet-like BiVO_4_ CB to MIL-125(Ti) CB while the holes would migrate from the MIL-125(Ti) VB to platelet-like BiVO_4_ VB and this would provide a spatial separation of the electrons and holes, which is propitious for improved photocatalytic activity. Literature reports that MIL-125(Ti) has a photochromic behaviour which is linked to the existence of intervalence electron transfer bands as a result of the optically induced movement of electrons to form Ti^3+^ from Ti^4+^ sites in the Ti oxo-clutters of MIL-125(Ti)^28^. Since MIL-125(Ti) cannot be excited by visible light but BiVO_4_ can, the visible light excited electrons in the BiVO_4_ are transferred via the intervalence electron transfer bands to reduce Ti^4+^ to Ti^3+^ in the Ti oxo-cluster in the MIL-125(Ti)^29^. Furthermore, the changes of the MIL-125(Ti) energy levels was perhaps caused by the alignment of the intervalence electron transfer bands to the fermi levels of BiVO_4_, that are closer to the conduction band, when the Schottky contacts are formed. The intervalence electron transfer mechanisms are shown in Equation (4, 5). The band positions of the pristine MIL-125(Ti) and platelet-like BiVO_4_ are shown in Figure S7.

4 $${{\rm{BiVO}}}_{4}+hv\to {{\rm{BiVO}}}_{4}({{\rm{e}}}^{-}+{{\rm{h}}}^{+})$$

5 $${\rm{MIL}}-125({\rm{Ti}})+{{\rm{e}}}^{-}\to {{\rm{Ti}}}^{3+}-{\rm{MIL}}-125({\rm{Ti}})$$

**Figure 8:**
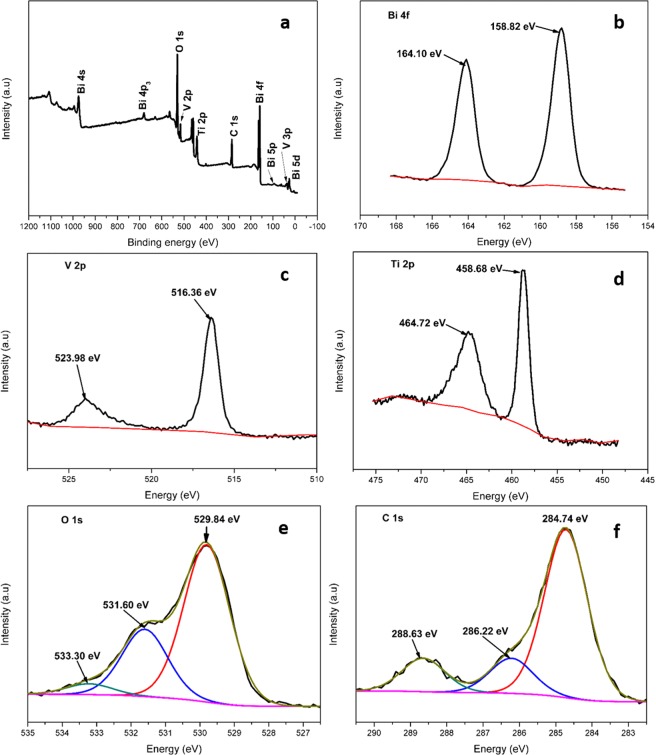
The XPS spectra of 3-2 Bi:Ti; (**a**) full survey spectrum, (**b**) Bi 4 f spectrum, (**c**) V 2p spectrum, (**d**) Ti 2p spectrum, (**e**) O 1 s spectra and (**f**) C 1 s spectra.

To ascertain the elemental constitution, chemical state and further confirm the formation of the heterojunction, XPS analysis was carried out. Figures 8a–f (3-2 Bi:Ti) depicts the XPS spectra together with the deconvoluted XPS peaks of Bi, V, Ti, O while the BiVO_4_ survey spectrum is shown in Figure S8. The survey spectrum depicted in Fig. 8a indicates the presence of O, V, Ti, C and Bi in the 3-2 Bi:Ti composite. The Bi 4 f spectrum in Fig. 8b displays peaks centred at 164.90 eV and 159.60 eV attributable to Bi 4f_5/2_ and Bi 4f_7/2_.respectively. The difference between the binding energies is 5.28 eV confirming the existence of Bi^3+^ in the composite^36^. The regional spectrum of V in Fig. 8c presents two peaks at 517.20 eV and 524.10 eV attributed to V 2p_3/2_ and V 2p_1/2_ which indicates that vanadium in the composite exists in +5 oxidation state^37^. Figure 8d depicts the Ti 2p spectra of Bi-Ti. The binding energy values of Ti 2p_3/2_ and Ti 2p_1/2_ at 458.80 and 465.90 eV correspondingly indicating that titanium bounded to oxygen exists as Ti^4+^ for titanium -oxo-cluster^26,29^. The spectrum of O1s is presented in Fig. 8e and was deconvoluted into three peaks attributed to oxygen from the Ti–O bond in the titanium-oxo-cluster at 529.84 eV, oxygen in hydroxyl groups at 531.60 eV and oxygen atoms in carboxyl groups at 533.30 eV^22^. Figure 8f displays the spectra of C1s which was resolved into three peaks ascribed to C–O at 284.74 eV and 286.22 eV, and O–C=O at 288.63 eV. These were attributed to the MIL-125(Ti) framework, which confirmed that MIL-125(Ti) was formed instead of TiO_2_.

**Figure 9:**
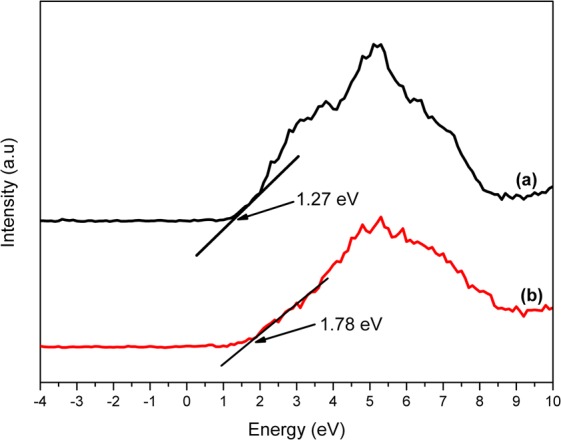
The VBs spectra of (**a**) BiVO_4_ and (**b**) 3-2 Bi:Ti.

These results conclusively validate the formation of a heterojunction between MIL-125 and BiVO_4_. Further proof was observed on the XPS VBs data that showed a more positive shift of the 3-2 Bi:Ti (1.78 eV) VB when compared to the BiVO_4_ VB of 1.27 eV (Fig. 9). The data firmly affirmed that the resulting heterojunction would be more suited for the photodegradation of organics in water or in oxygen production in water-splitting. As observed from the qualitative calculation of the VB potentials using DRS, the heterojunction was expected to show minimum recombination, and this was confirmed by the XPS data which gives more precise numerical values of the valence band edge potential of the materials.

**Figure 10:**
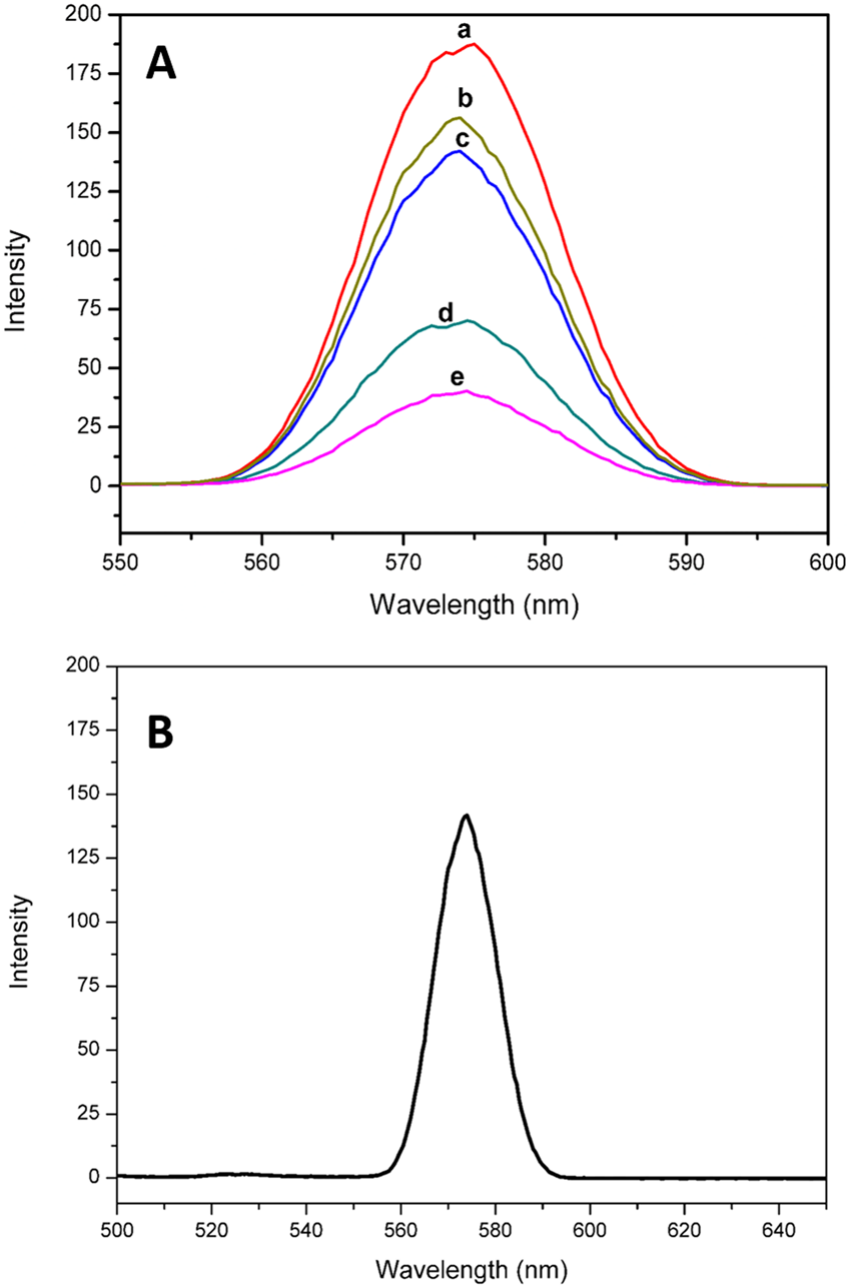
(**A**) PL measurements of (a) platelet-like BiVO4, (b) 3-2 Bi:Ti (PM), (c) 1–1 Bi:Ti, (d) 2–3 Bi:Ti and (e) 3-2 Bi:Ti. (**B**) PL measurement of MIL-125(Ti).

Photoluminescence (PL) spectra of the NPs is depicted on Fig. 10A. Data from the PL yields information about the electronic properties of the NPs, whereby the intensity of the graphs gives an insight about the recombination rate of photogenerated charge carriers in the NPs. This is specifically about the availability of surface states and appropriate data about the capability of NPs to trap charges. To correlate PL and mitigation of recombination in photoactive materials, reported in literature is that decreased intensity shows less recombination of photogenerated charge carriers^38^. Thus, there would be inhibited recombination in 3-2 Bi:Ti, followed by 2–3 and 1–1 Bi:Ti shown by Fig. 10A (d), (c) and (b) respectively. The highest recombination was observed for platelet-like BiVO_4_. The MIL-125(Ti) has an overlapping intensity to that of 1–1 Bi:Ti composite which justifies why it was plotted separately in Fig. 8B. This inferred comparable recombination rates for the materials. Perhaps the quality of the Schottky contact is not favourable enough to effectively facilitate the separation of the electrons and holes in 1–1 Bi-Ti. Furthermore, a physically mixed composite of BiVO_4_ and MIL-125(Ti) (3-2 Bi:Ti (PM)) was fabricated to investigate the quality of the Schottky contact formed (Fig. 10A). The physically mixed intermediate exhibited a strong peak compared to the heterojunction intermediates indicating that there was no interface formed when the materials were physically mixed as a high recombination rate translates to poor separation efficiency of electrons and holes.

**Figure 11:**
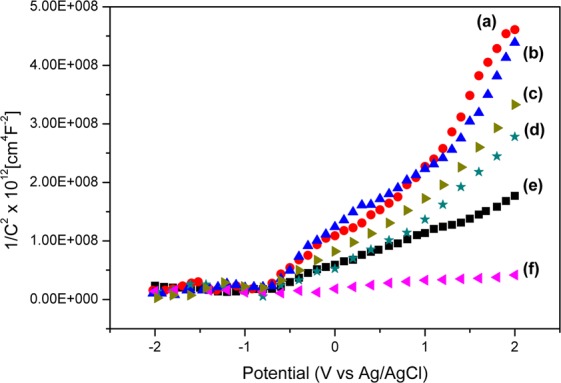
Mott-Schottky plots of (**a**) platelet-like BiVO_4_, (**b**) MIL-125(Ti), (**c**) 3-2 Bi:Ti (PM), (**d**) 1–1 Bi:Ti, (**e**) 2–3 Bi:Ti and (**f**) 3-2 Bi:Ti.

As deduced from the potentiostat, Mott-Schottky plots were plotted through Equation (6) to understand the result of heterostructure formation.

6 $$\frac{1}{{C}^{2}}=\frac{2}{\varepsilon {\varepsilon }_{0}{e}_{0}{N}_{D}}(E-{E}_{FB}-\frac{kT}{{e}_{0}})$$

where *ε* is semiconductor electrode permittivity, *e*_*0*_ is elementary charge, *N*_*D*_ is donor density, *E* is applied potential, *E*_*FB*_ is flat-band potential, *k* is Boltzmann constant, T is operation temperature and *C* is capacitance of space charge. The Mott-Schottky plots are shown in Fig. 11 and they presented good correlation to the PL data. The given slope computed from 1/C^2^ against *E* according to Equation (6) which was further used to observe recombination rates of the NPs. A decreased slope after the formation of the heterojunctions signifies reduced recombination rates of electrons and holes^15^. As observed, the recombination rate was in the following order; platelet-like BiVO_4_ > MIL-125(Ti) > 1–1 Bi:Ti > 2–3 Bi:Ti > 3-2 Bi:Ti which compared well to the PL data (Fig. 10). The physically mixed (PM) composite (3-2 Bi:Ti (PM)) had a slope lower than that of the pristine materials but higher than that of the composite which further showed that the hydrothermally synthesised heterojunctions successfully formed Schottky contacts that lead to the separation of photoinduced charge carriers. All the NPs slopes that are positive, and this is typical to *n*-type semiconductors. Thus, both platelet-like BiVO_4_ and MIL-125(Ti) are *n*-type semiconductors.

**Figure 12:**
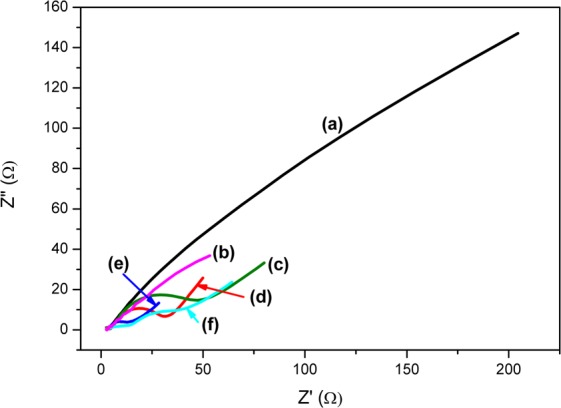
Nyquist plots of (**a**) platelet-like BiVO_4_, (**b**) 3-2 Bi:Ti (PM), (**c**) MIL-125(Ti), (**d**) 1–1 Bi:Ti, (**e**) 2–3 Bi:Ti and (**f**) 3-2 Bi:Ti.

Electrochemical impedance spectroscopy (EIS) in the form of Nyquits plots was used to investigate the interfacial phenomena that happens between the electrolytes and the photoelectrode. Interpretation of EIS done through the Nyquist plots gives differences in recombination rates by studying the diameters of semicircles formed between real (Z’ (Ω)) parts and imaginary (Z”(Ω)) parts of complex impedance. This then translates to a larger semicircle diameter signifying larger charge transfer resistance of the working electrode thus higher recombination rates. The Nyquist plot may in some cases have a semicircle that joins to another larger semicircle or nonvertical line at intermediate frequencies. The second semicircle or nonvertical line gives insights on the electrode roughness. The initial semicircle is ascribed to the charge transfer resistance or electrolyte resistance. The larger semicircle is atributed to limitation of ion transport in the electrolyte for porous electrode structures^39^. Figure 12 then illustrates the synthesized NPs’ Nyquist plots. The charge resistance was observed to decrease in the order platelet-like BiVO_4_ > MIL-125(Ti) > 1–1 Bi:Ti > 2–3 Bi:Ti > 3-2 Bi:Ti which confirmed the information observed from the PL data and Mott-Schottky plots. Thus, the optical and electrochemical characterization data showed a successful formation of *n*-*n* junctions between platelet-like BiVO_4_ and MIL-125(Ti) with the optimum being at 3-2 Bi:Ti. This was further proved by the physically mixed composite 3-2 Bi:Ti (PM) which had a high recombination rate compared to the hydrothermally sythesized heterojunctions shown by its larger larger semicircle. This signifies that the formation of hetero-interfaces enhances the separation of photoinduced charge carriers thus inhibiting recombination.

## Conclusion

Different BiVO_4_-MIL-125(Ti) with diverse molar ratios of Bi:Ti were synthesized through a two-step hydrothermal synthesis protocol. The results revealed that the different heterojunctions were successfully synthesized. Confirmation of the formation of the heterojunction was substantiated by the XPS data which showed a positive shift of the VB in the 3-2 Bi:Ti photocatalyst. The shift also shows that the heterojunction formed would be more suited for the degradation of organics in water as a more positive VB favours the photodegradation of organics in water. Furthermore, the optical and photoelectrochemical studies showed that the photocatalyst 3-2 Bi:Ti exhibited the slowest recombination rate and the fastest interfacial charge shuttle permitted by the *n*-*n* junction formed. The synthesis of BiVO_4_-MIL-125(Ti) heterojunction can possibly expand the scope of semiconductor-MOF heterojunctions with expected new properties and applications.

## Supplementary information

A composite of platelet-like orientated BiVO4 fused with MIL-125(Ti): Synthesis and characterization
