# Supplementary material for: A composite of platelet-like orientated BiVO4 fused with MIL-125(Ti): Synthesis and characterization
Source: Sci Rep. 2019 Jul 11;9:10044. doi: 10.1038/s41598-019-46498-w (PMC6624298; doi:10.1038/s41598-019-46498-w)
Supplement: Supplementary file 1 — A composite of platelet-like orientated BiVO4 fused with MIL-125(Ti): Synthesis and characterization [file 41598_2019_46498_MOESM1_ESM.pdf]

Supplementary Information

A composite of platelet-like orientated $BiVO_4$ fused with MIL-125(Ti): Synthesis and characterization

Philani Vusumuzi Hlophe, Lwazi Charles Mahlalela and Langelihle Nsikayezwe Dlamini*

Department of Chemical Sciences, University of Johannesburg, Doornfontein Campus, P.O. Box 17011, Doornfontein, Johannesburg, 2028, South Africa

**Corresponding Author; Email: lndlamini@uj.ac.za, Tel: (+27) 011 559 6945*

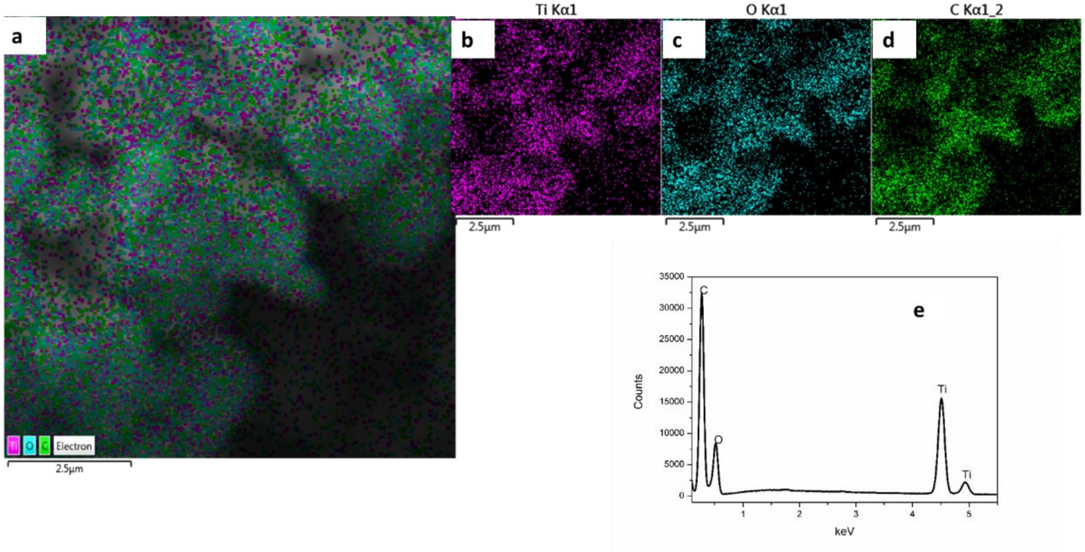

Figure SI SEM mapping image of (a) MIL-125(Ti) with respective inserts of (b) Titanium (c) Oxygen and (d) Carbon. (e) EDS of mapped image

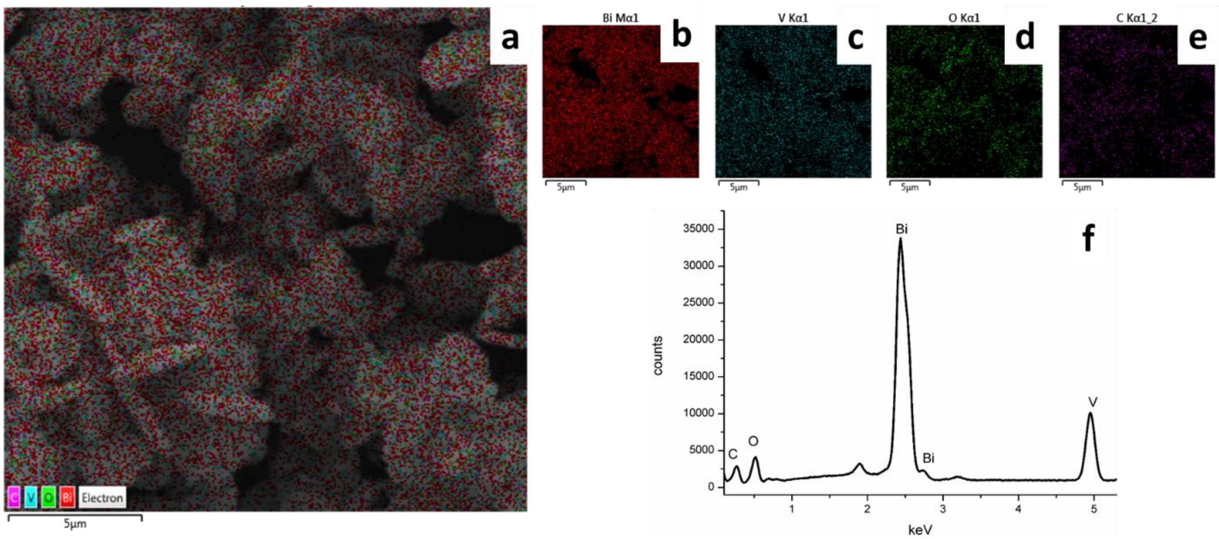

Figure S2 SEM mapping image of (a) $BiVO_4$ with respective inserts of (b) Bismuth (c) Vanadium (d) Oxygen and (e) Carbon. (f) EDS of mapped image

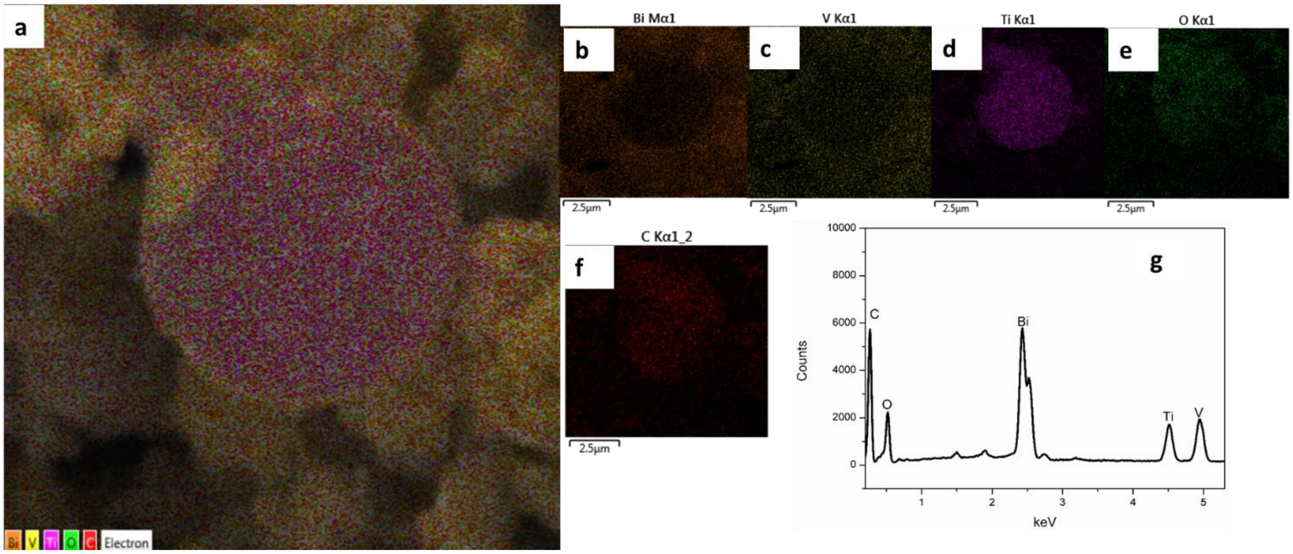

Figure S3 SEM mapping image (a) 1-1 Bi:Ti composite with respective inserts of (b) Bismuth (c) Vanadium (d) Titanium (e) Oxygen and (f) Carbon. (g) EDS of mapped image

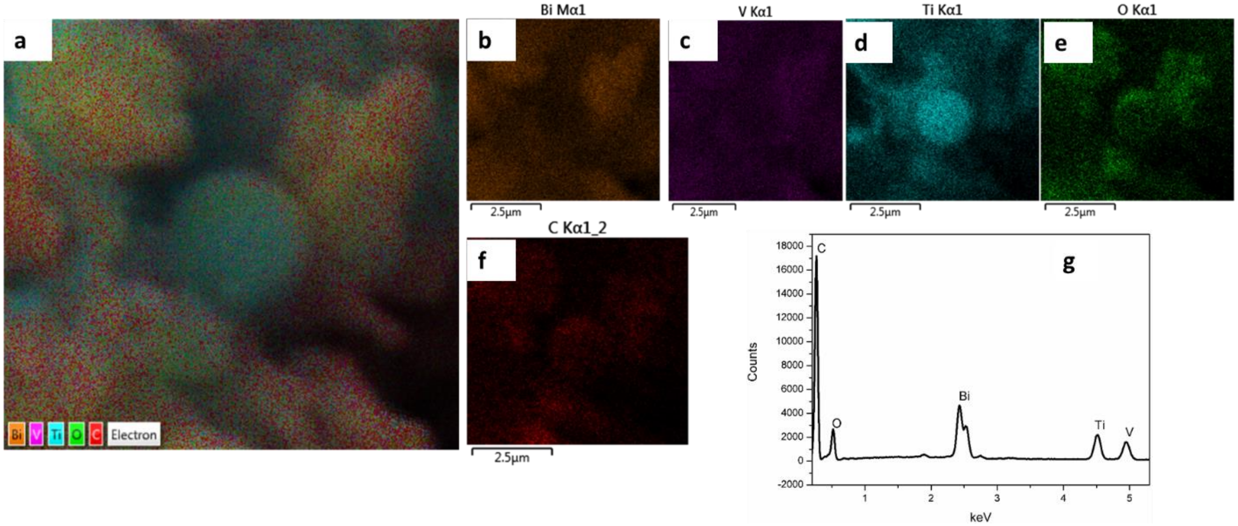

Figure S4 SEM mapping image (a) 2-3 Bi:Ti composite with respective inserts of (b) Bismuth (c) Vanadium (d) Titanium (e) Oxygen and (f) Carbon. (g) EDS of mapped image

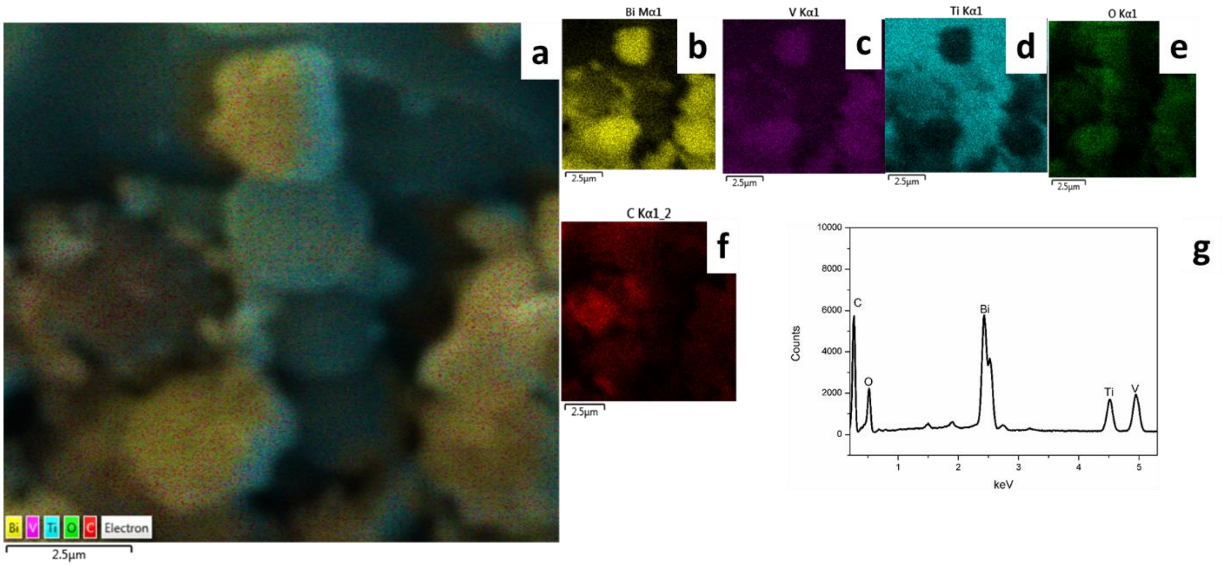

Figure S5 SEM mapping image (a) 3-2 Bi:Ti composite with respective inserts of (b) Bismuth (c) Vanadium (d) Titanium (e) Oxygen and (f) Carbon. (g) EDS of mapped image

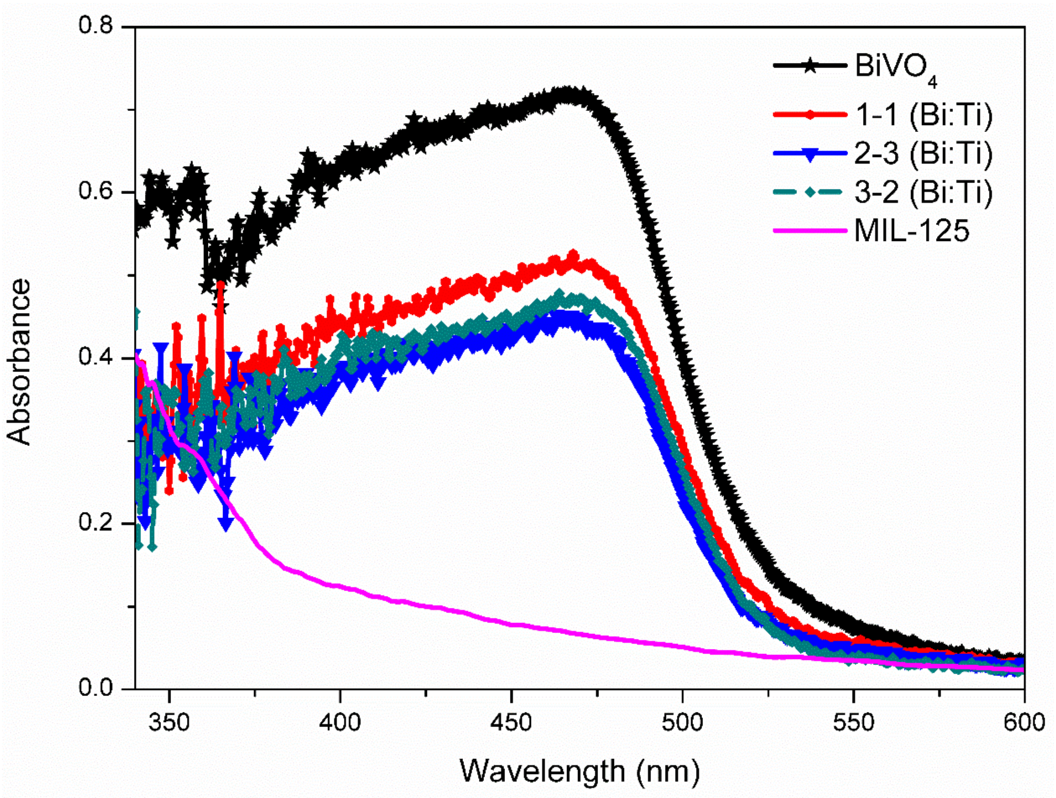

Figure S6 UV-Vis spectra of MIL-125(Ti), $BiVO_4$ and Bi:Ti composites

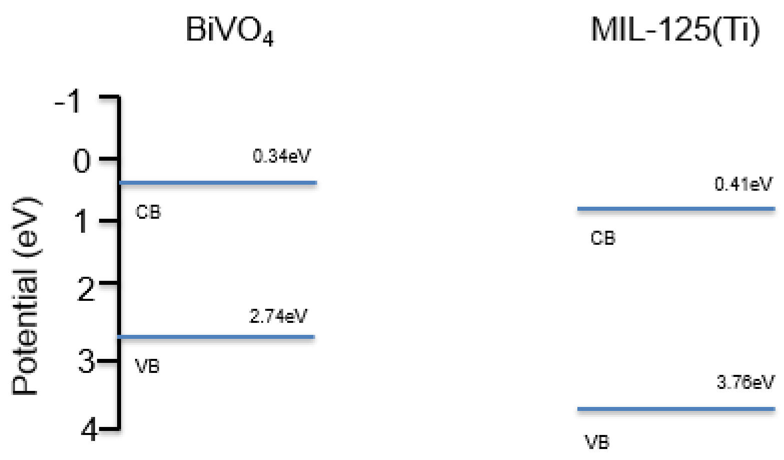

Figure S7 Band positions of $BiVO_4$ and MIL-125(Ti)

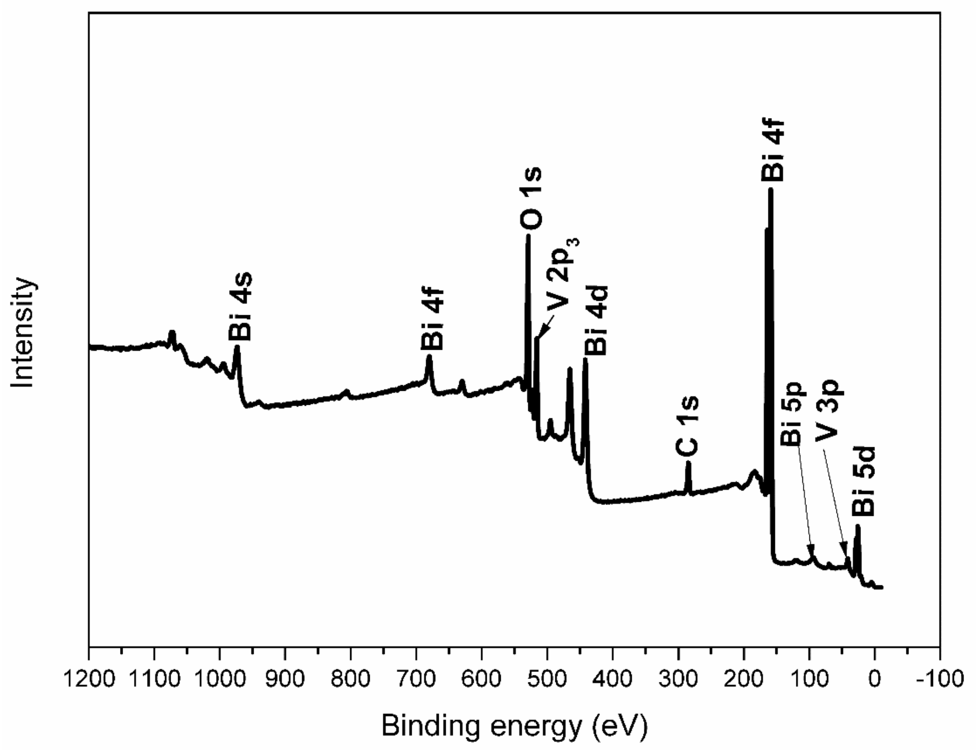

Figure S8 The XPS full survey spectrum of $BiVO_4$
